# Fabry nephropathy: a review – how can we optimize the management of Fabry nephropathy?

**DOI:** 10.1186/1471-2369-15-72

**Published:** 2014-05-06

**Authors:** Stephen Waldek, Sandro Feriozzi

**Affiliations:** 1 31 Harboro Road, Sale, Cheshire M33 5AN, UK; 2 Department of Nephrology and Dialysis, Belcolle Hospital, Viterbo, Italy

**Keywords:** Fabry disease, Diagnosis, Management, Nephropathy, Enzyme replacement therapy

## Abstract

Fabry disease is a rare, X-linked, lysosomal storage disease caused by mutations in the gene encoding the enzyme alpha-galactosidase A. Complete or partial deficiency in this enzyme leads to intracellular accumulation of globotriaosylceramide (Gb_3_) and related glycosphingolipids in many cell types throughout the body, including the kidney. Progressive accumulation of Gb_3_ in podocytes, epithelial cells and the tubular cells of the distal tubule and loop of Henle contribute to the renal symptoms of Fabry disease, which manifest as proteinuria and reduced glomerular filtration rate leading to chronic kidney disease and progression to end-stage renal disease. Early diagnosis and timely initiation of treatment of Fabry renal disease is an important facet of disease management. Initiating treatment with enzyme replacement therapy (ERT; agalsidase alfa, Replagal^®^, Shire; agalsidase beta, Fabrazyme^®^, Genzyme) as part of a comprehensive strategy to prevent complications of the disease, may be beneficial in stabilizing renal function or slowing its decline. Early initiation of ERT may also be more effective than initiating therapy in patients with more advanced disease. Several strategies are required to complement the use of ERT and treat the myriad of associated symptoms and organ involvements. In particular, patients with renal Fabry disease are at risk of cardiovascular events, such as high blood pressure, cardiac arrhythmias and stroke. This review discusses the management of renal involvement in Fabry disease, including diagnosis, treatments, and follow-up, and explores recent advances in the use of biomarkers to assist with diagnosis, monitoring disease progression and response to treatment.

## Review

Fabry disease is a rare, X-linked, lysosomal storage disease (OMIM #301500) caused by mutations in the gene encoding the acid hydrolase enzyme alpha-galactosidase A (EC 3.2.1.22), which catalyses removal of a galactose moiety from neutral sphyngolipids, predominantly globotriaosylceramide (Gb_3_). Deficiency in this enzyme causes intracellular accumulation of Gb_3_ and related glycosphingolipids in a wide range of cell types throughout the body [1]. The major clinical manifestations include pain in the hands and feet (acroparaesthesia), angiokeratoma, as well as renal, cardiac, and cerebrovascular disease [2].

The Fabry Outcome Survey (FOS; sponsored by Shire) [2], and Fabry Registry (sponsored by Genzyme) [3-5] are databases established to collect longitudinal data on Fabry disease. These databases have shown a long delay between the onset of initial symptoms and a diagnosis. For example, within the Fabry Registry, median ages for initial onset of symptoms and a diagnosis were 9 and 23 years in males, and 13 and 32 years in females, respectively [3]. Many paediatric patients (aged <19 years) with Fabry disease report early symptoms. At enrolment in the Fabry Registry (median age, 12 years; n = 352), 77% of males and 51% of females had symptoms or signs of Fabry disease, including some patients who had already developed stage-2 or -3 chronic kidney disease (CKD; n = 3) [4]. Assessment of the renal morphological changes in Fabry disease using light microscopy and electron microscopy in 9 symptomatic paediatric patients (7 males, 2 females; 7–18 years) revealed glomerular and vascular changes despite normal renal function [6]. The combination of acroparasthesia, mild albuminuria, glomerular endothelial cell deposits, and arteriopathy may indicate a more severe phenotype [6].

Classically affected males with Fabry disease develop overt proteinuria and progressive renal impairment by the second-to-fifth decades of life [5]. Females with Fabry disease also have a significant risk of major organ involvement. Among adult females with estimated glomerular filtration rate (eGFR) data (n = 638), 62.5% had an eGFR <90 ml/min/1.73 m^2^ and 19.0% had an eGFR <60 ml/min/1.73 m^2^[5]. Approximately 30–35% of females with Fabry disease have proteinuria [7,8], 13% have stage 3 CKD [9] and 1–4% have end-stage renal disease (ESRD) [5,10].

A wide range of renal histopathology is found in Fabry disease [11]. All cell types within the kidney are affected, even in patients with normal GFR and minimal proteinuria [12]. Vacuolization of podocytes and epithelial cells is characteristic of Fabry disease, with mesangial expansion and progressive segmental and global glomerulosclerosis also observed [12-15]. It is becoming increasingly clear that podocytes have an important role in proteinuria. In a study of renal histology conducted prior to enzyme replacement therapy (ERT) in 14 young patients (age range: 4–19 years), podocyte Gb_3_ inclusion volume and density was shown to increase with age [16]. Podocyte foot process width also increased with age and was directly correlated with proteinuria. In the same study, endothelial fenestrations were reduced, indicating a central role for podocytes in the development and progression of Fabry nephropathy [16]. Additionally, accumulation of Gb_3_ in tubular cells in the distal tubule and loop of Henle may also contribute to some of the early renal symptoms of Fabry renal disease [17,18].

**Figure 1:**
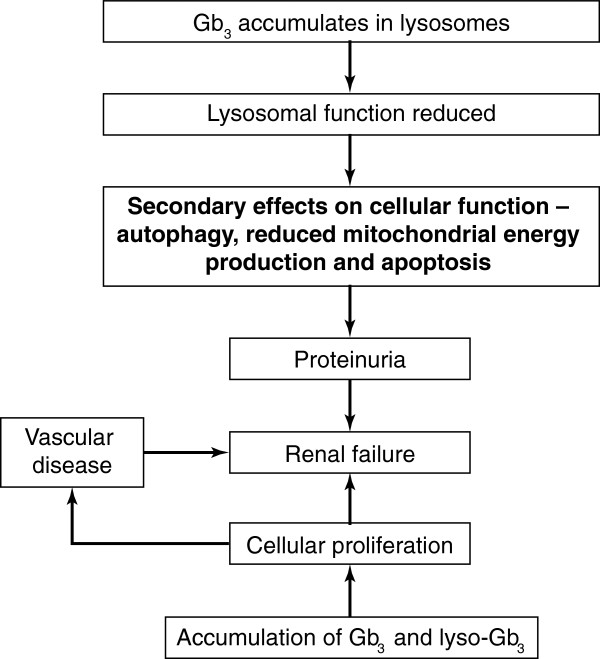
**Pathogenesis of Fabry nephropathy. **Gb_3_, globotriaosylceramide; lyso-Gb_3_, globotriaosylsphingosine.

The pathogenesis of Fabry renal disease is not fully understood and is likely to be multifactorial (Figure 1). Progressive intracellular accumulation of Gb_3_ leads to cellular changes and histological damage. Deposition of Gb_3_ can promote vascular smooth muscle cell proliferation and the release of mediators involved in the pathogenesis of other nephropathies [19]. Other factors considered to be involved in the pathophysiology are the rupture of the lysosome [20] and damage due to local inflammation [21]. Furthermore, addition of Gb_3_ to cultured endothelial cells has been found to increase oxidative stress as demonstrated by an increase in reactive oxygen species and up-regulation of the expression of cell adhesion molecules [22]. Vasculopathy has also been observed in patients with Fabry disease that may be due to the local up-regulation of the renin–angiotensin system (RAS) [23].

Glomerular sclerosis and tubulo-interstitial fibrosis are the histological features that best correlate with the progression of renal disease in humans with Fabry disease [24]. As already mentioned, accumulation of Gb_3_ in podocytes plays an important role in the pathogenesis of glomerular damage. A human podocyte model of Fabry disease was developed using RNA interference to reduce alpha-galactosidase A expression. Accumulation of Gb_3_ was accompanied by an increase in autophagosomes, suggesting that deregulated autophagy pathways have some involvement in the pathogenesis of glomerular damage in Fabry disease [25].

Progression to ESRD results in the need for renal replacement therapy (RRT) [26]. The extra-renal manifestations of Fabry disease persist in patients receiving RRT [27]. Renal failure increases the risk of cardiovascular events and, consequently, those who develop ESRD are at a greater risk of experiencing cardiovascular events and strokes than patients with Fabry disease overall [8]. Indeed, cardiovascular disease is the most common cause of death in patients with Fabry disease [28].

## Diagnosis and assessment of Fabry nephropathy

The American College of Medical Genetics and the National Society of Genetic Counselors have issued guidelines on the diagnostic confirmation of lysosomal storage disorders, including Fabry disease [29]. Enzymatic analyses of dried blood spots allow population screening and an initial diagnosis in males [30], whilst in heterozygous females, in whom alpha-galactosidase A activity is highly variable, genotyping is essential for a diagnosis. Genotyping can also be useful in male patients to assist with tracing family history of the disease. Urine microscopy may be clinically useful in diagnosing Fabry disease: vacuolated epithelial cells, filled with glycosphingolipids, giving the appearance of a ‘Maltese cross’, can be seen using polarized light microscopy [31]. Assessment of renal Fabry disease involves several assessments and tools (Table 1), with renal biopsy being particularly important to assess and monitor disease progression long term [32]. It has been reported that globotriaosylsphingosine (lyso-Gb_3_), a product of Gb_3_ metabolism, is elevated in male patients with Fabry disease and that it can be a useful marker in secondary screening to differentiate mutations of the alpha-galactosidase A gene causing Fabry disease from those not causing disease [33].

**Table 1:** Recommendations for the management of Fabry renal disease in adults

	**Recommendations**
**Diagnosis and assessments**	
Confirm diagnosis	• Confirm presence of Fabry disease (by enzyme analysis in males and by gene mutation studies in females)
	• GFR <90 ml/min/1.73 m^2^ (CKD stage 1–5)
	• Proteinuria: >30 mg/day or >30 mg/g creatinine (albuminuria); >300 mg/day or >300 mg/g creatinine (proteinuria)
	• Other renal conditions excluded rigorously (even if a renal biopsy is needed to make that exclusion)
Kidney biopsy	• Histological injury can precede clinical signs, and provides a compelling indication for institution of ERT, especially in children and young adults
	• Excludes other conditions (especially in patients with atypical presentations)
	• Confirms the diagnosis and stage and can be used to assess response to therapy
Initial assessment and follow-up	• Measure serum creatinine and use CKD-EPI equation to estimate the GFR
	• Use iohexol plasma clearance or isotopic methods (depending on local availability) for precise measurement of the GFR if the eGFR >60 ml/min/1.73 m^2^
	• Standard CKD assessment schedule
	• Quantify urinary albumin and protein levels
	• Calculate eGFR slope
**Treatment**	
ERT	• Agalsidase alfa or beta at approved dose
	• Start ERT as soon as the definitive diagnosis has been made in patients with little or no residual enzyme activity
	• Start ERT as soon as the definitive diagnosis has been made in patients with residual enzyme activity if there is evidence of kidney involvement
	• ERT will not reduce proteinuria (in adults)
Control of proteinuria	• Use ACE inhibitors and/or ARBs in addition to ERT
	• Titrate doses to achieve urine protein <500 mg/day, even if blood pressure <130/180 mmHg
	• Effects on progression are likely to occur only in the setting of optimal ERT dosing
Other therapy	• All other aspects of standard CKD care apply to the management of Fabry renal disease

*ACE*: angiotensin-converting enzyme; *ARB*: angiotensin-receptor blocker; *CKD*: chronic kidney disease; *CKD-EPI*: Chronic Kidney Disease Epidemiology Collaboration; *eGFR:* estimated glomerular filtration rate; *ERT*: enzyme replacement therapy; *GFR*: glomerular filtration rate.

Adapted and republished with permission of American Society of Nephrology, from [Enzyme replacement therapy and Fabry renal disease. Warnock DG et al. Clin J Am Soc Nephrol 5: 2010]; permission conveyed through Copyright Clearance Centre, Inc. [34].

A new classification for Fabry disease has recently been proposed based on the functional characterization of *GLA* mutations. Lucas *et al.* correlated enzyme activity levels with specific mutational effects on the enzyme’s three-dimensional structure and the resulting alteration in function. It has been proposed that this classification will facilitate the diagnosis of Fabry disease, especially when combined with biomarker data such as lyso-Gb_3_ levels [35]. The proposed system of classification may help with the so-called late onset single organ phenotypes, some of which may not be clinically insignificant.

### Progression of renal disease

In Fabry disease, the decline in renal function over time is related to the degree of proteinuria and, in untreated patients, is more rapid when the eGFR is below 60 ml/min/1.73 m^2^. Male sex and hypertension are also significant risk factors for development of renal failure [36]. As with all nephropathies, protein overload may cause an increase in the levels of inflammatory mediators, and interstitial accumulation of these mediators may lead to renal scarring. In patients with undiagnosed Fabry renal disease, a significant number of glomeruli may already be sclerotic [37]. Reduced nephron mass thus increases the risk of further renal damage from hyperfiltration, proteinuria, and activation of angiotensin II [37].

Consequently, regular, reproducible estimates of renal function are essential in the management of Fabry disease. Most centres now use the eGFR calculated using the MDRD (modified diet in renal disease) equation to assess renal function during follow-up [38]. There are limitations to current GFR estimates [39] and the CKD-EPI (Chronic Kidney Disease Epidemiology Collaboration) equation is recommended in adults. In children, a CKD-EPI equation developed by Schwartz et al. has been used successfully [40]. GFR can be measured more accurately using the iohexol plasma disappearance curve [40]. Rombach and colleagues suggest that the Stevens’ equation (a creatinine- and cystatin C-based formula) is also helpful, although it is recognized that cystatin C is not uniformly available [39].

### Proteinuria

Proteinuria is an early sign of Fabry renal disease in both sexes and is often the most frequent clinical manifestation [5,6]. Importantly, proteinuria is an independent risk factor affecting the extent of renal decline in treated and untreated patients, as well as determining the success of ERT [41-44].

Among patients in the FOS, proteinuria was present in 44–54% of males and 33–41% of females for whom renal data were available [45]. Similarly, data from 1,262 adult patients (585 males, 677 females) in the Fabry Registry demonstrated overt proteinuria (>300 mg/day) in 43% and 26% of males and females with CKD stage 1, respectively, with higher proportions in patients with more advanced kidney involvement [8]. Consequently, proteinuria should be monitored regularly and treated appropriately.

### Renal biopsy

Renal biopsy is useful in all patients with any degree of proteinuria/albuminuria and/or renal dysfunction to assess the degree of glomerulosclerosis and interstitial damage because of their prognostic significance [2]. In patients with minimal proteinuria and normal renal function, biopsy can also determine if there is significant Gb_3_ deposition (especially in podocytes and endothelial cells), or early damage to indicate ERT use. In the classical male patient with signs of renal involvement a renal biopsy can be helpful in providing evidence of prognosis depending on the degree of glomerular sclerosis [41]. In females with even the slightest evidence of Fabry nephropathy, renal biopsy is considered much more important (and by some mandatory) as the presence of significant renal Gb_3_ deposits is an indication for initiating ERT. Renal biopsy should also be undertaken if there is the possibility of double pathology (e.g. diabetes or other glomerular diseases such as immunoglobulin A [IgA] nephropathy or thin membrane disease if haematuria is present) and if there is a sudden, unexplained decline in renal function [46,47].

**Figure 2:**
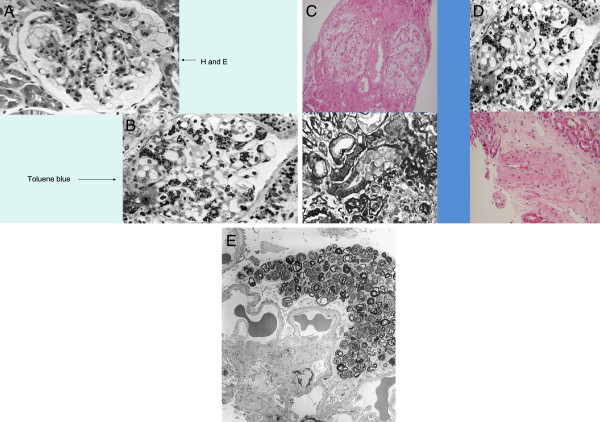
**Histological diagnosis of Fabry disease. (A)** Haematoxylin and eosin stain of tissue from a patient with Fabry disease showing lesions that could be confused with other conditions in which foam cells are present; **(B)** Semi-thin section stained with toluene blue (the preferred method for diagnosing Fabry disease on renal histology); **(C)** deposits in the tubules that can give rise to mild renal tubular disorders; **(D)** a small artery with large degrees of narrowing due to Gb_3_ deposits; **(E)**  ultrastructural image (electron microscopy) of the glomerulus in Fabry disease shows lamellated lipid inclusions (zebra bodies) within the podocyte cytoplasm (×6,000; stain: UrPb). Reproduced with the permission of Dr. Kostas Giannakakis (Laboratory of Ultrastructural Pathology, Rome La Sapienza University, Rome, Italy). E, eosin; H, haematoxylin; UrPb, uranyl lead.

Fabry disease can be difficult to diagnose solely from standard light microscopy, so semi-thin toluene blue sections or electron microscopy should be performed particularly if there is doubt about the histological diagnosis [13]. The role of re-biopsy is still undetermined, especially for evaluating the results of ERT. However, a study by Tondel et al. found comparing deposition of Gb_3_ in glomerular cells from renal biopsies taken at baseline and after 5 years of ERT to be useful in assessing the success of ERT [32]. It is better to compare Gb_3_ deposition using semi-thin toluene-blue stained sections and/or electron microscopy, rather than with haematoxylin and eosin or periodic acid Schiff stained slides, where the Gb_3_ deposition is dissolved by solvents during processing of the samples. Figure 2A illustrates how foam cells observed with haematoxylin and eosin staining could be confused with other causes of glomerular nephritis. Typical Fabry lesions observed with toluene-blue staining or electron microscopy are shown in Figures 2B–E.

### Renal ultrasound and haematuria

Multiple renal cysts, mostly parapelvic, can be detected using ultrasonography in up to 50% of cases of patients [48]. Although uncommon, haematuria may also occur, but other causes must be excluded [46,47]. The aetiology of cysts is unknown, but the cause of haematuria is similar to that found in other glomerular diseases.

### Hypertension

In the FOS Registry, a high prevalence (57% of male and 47% of female patients) of uncontrolled hypertension was reported in patients with Fabry disease which generally increased with worsening CKD stage [49]. Similarly, in the Fabry Registry, hypertension (systemic blood pressure [BP] ≥130/80 mmHg) was observed more often in those with poor renal function (67% vs 48% of patients with eGFR ≥60 ml/min/1.73 m^2^) [8].

In general, the BP of patients with Fabry disease is lower than that of the general population, predominantly due to the effects of autonomic dysfunction on cardiac and vascular function [50], although the aetiology of this effect is complex and other factors may be involved. Consequently, the current target for controlling hypertension in the general population (systolic BP <130 mmHg or diastolic BP <80 mmHg) may be too high. Maintenance of a BP <125/75 mmHg in patients with proteinuria levels >1 g/day, and <130/80 mmHg in patients with proteinuria of 0.25–1 g/day has been advocated in CKD [37]. Caution is recommended in reducing BP <100/70 mmHg because this has been shown to increase the risk of cardiovascular and/or renal events [31,51,52].

## Management of Fabry renal disease

### ERT in Fabry disease

Two ERT products are available for treating Fabry disease: agalsidase alfa (Replagal^®^, Shire), produced in a human cell line by gene activation technology [53], and agalsidase beta (Fabrazyme^®^, Genzyme), produced in Chinese hamster ovary (CHO) cells [54]. The recommended licensed dose for agalsidase alfa is 0.2 mg/kg/2 weeks and is 1 mg/kg/2 weeks for agalsidase beta.

Current UK guidelines for the management of Fabry disease state that, “*No trial has yet addressed the appropriate starting time of treatment or the group of patients most likely to benefit from therapy. However this is a chronic, progressive disorder. The aim of treatment is to prevent progression and where disease is already manifest to try and reverse or stabilise the disease. It is anticipated that treatment will be most successful when started early in the course of the disease. Conversely treatment late in the course of the disease may have limited efficacy.”*[38]. Therefore ERT should be started as soon as symptoms/signs occur in an effort to stabilize (or even reverse) pathology. In the UK, ERT treatment is indicated in patients with Fabry disease who have evidence of renal disease (defined as an eGFR <80 ml/min, proteinuria >300 mg/day) and/or microalbuminuria if a renal biopsy shows endothelial deposits [38,51].

ERT has been shown to improve the clinical outcome of patients with Fabry disease, including stabilization of kidney function and reduction in neuropathic pain [41,55-57]. However, because of the heterogeneous disease presentation, no evidence is available to support the optimal timing of ERT or to identify the patient groups in whom disease is likely to be most rapidly progressive or which patients are most likely to gain significant benefit from therapy [58]. Data from the FOS have been used to develop an age- and sex-adjustment of the global Mainz Severity Score Index for Fabry disease. This scoring system corrects for the impact of age and sex on severity. By allowing meaningful comparisons across age and sex [59] it may be possible to determine clinical features that predict future disease severity.

Data from the FOS have also been used to develop organ-specific (cardiac, renal, and neurological) and composite prognostic severity scores [58]. Using the analysis it was possible to differentiate groups of patients with different outcome probabilities. The overall composite score, the Fabry International Prognostic Index (FIPI), distinguished two distinct groups in whom the 50% event-free survival differed by 10 years. The FIPI should prove to be a valuable tool in comparative analyses of outcomes using different therapies [58]. Additionally, a validated disease severity scoring system for Fabry disease has been developed (DS3) to quantify disease burden and monitor disease progression and response to treatment [60].

### Impact of ERT on renal function

Data on the renal effects of agalsidase alfa and beta mostly originate from open-label and observational studies, including the FOS and the Fabry Registry [41,45,51,55,61-64], with randomized, long-term, controlled data being limited to one placebo-controlled trial [44] and two studies that utilized historical controls [65,66]. Although registries can provide long-term data on large patient populations, they are limited by their voluntary nature, which can result in incomplete follow-up data [67].

### Short-term studies

The renal benefits of ERT at tissue and clinical levels were demonstrated in a double-blind, randomized, placebo-controlled study of 26 patients given agalsidase alfa at 0.2 mg/kg/2 weeks for 6 months [55]. Significant improvements were seen in renal structure (increase in the percentage of mesangium) in patients receiving agalsidase alfa compared with placebo [55].

Similarly, agalsidase beta 1 mg/kg/2 weeks resulted in stabilization of the GFR with rapid, marked, reductions in plasma and tissue Gb_3_ observed by biochemical and histological means [61]. A randomized, double-blind, placebo controlled Phase IV trial investigating the effects of agalsidase beta (1 mg/kg/2 weeks for up to 35 months [mean, 18.4 months]) on clinical outcomes in 82 adult patients with advanced Fabry disease and mild-to-moderate renal dysfunction found an increased time to first clinical event and stabilized renal function compared with placebo when adjusted for the degree of proteinuria [44].

Analyses of data from the FOS showed improvements in renal function based on 1,040 serum creatinine measurements in 201 patients [63]. Time on agalsidase alfa therapy was an independent predictor of serum creatinine levels (inverse association, p = 0.04; multivariate analyses). In patients with CKD stage 2 and 3, ERT halted the progressive decline in renal function within the first year of treatment (longitudinal analyses) [63].

### Long-term studies

The benefits of ERT with agalsidase beta over 54 months have also been reported: 41 patients with <50% glomerulosclerosis and ≤1 g proteinuria per 24 hours at baseline showed stable renal function [41].

**Figure 3:**
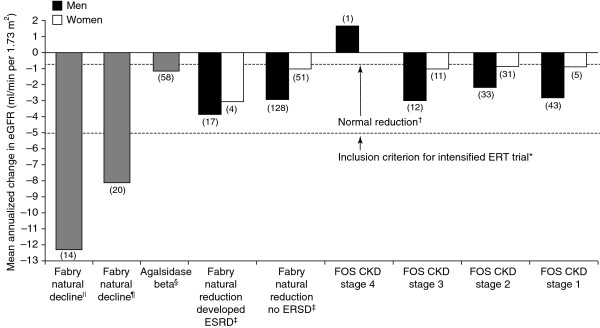
**Change in eGFR in patients with Fabry disease and CKD during treatment with agalsidase alfa.** Republished with permission of Elsevier, from [Enzyme replacement therapy with agalsidase alfa in patients with Fabry’s disease: an analysis of registry data. Mehta A et al. Lancet 374: 2009]; permission conveyed through Copyright Clearance Centre, Inc. [68]. Data are plotted according to baseline stage of CKD. Patient numbers are shown in parentheses. Data from previous studies for the expected natural fall in renal function in patients with Fabry disease and the effects of agalsidase beta are plotted for reference and comparison. *[69]; †[70]; ‡[18]; §[41]; ¶[63]; ||[26]. CKD, chronic kidney disease; GFR, glomerular filtration rate.

**Figure 4:**
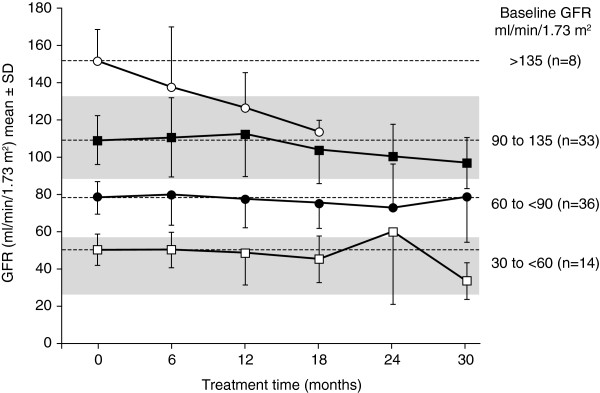
**GFR during agalsidase alfa treatment in male patients with Fabry disease (stratified by baseline GFR).** Republished with permission of American Society of Nephrology, from [Agalsidase alfa and kidney dysfunction in Fabry disease. West M et al. J Am Soc Nephrol 2009; 20: 2009]; permission conveyed through Copyright Clearance Centre, Inc. [56]. GFR, glomerular filtration rate.

Long-term positive results with ERT were confirmed in an analysis of renal function in a cohort of males and females (n = 150) in FOS receiving agalsidase alfa and adjunctive therapies for up to 5 years [68]. Annualized rates of decline by CKD stages are shown in Figure 3. Responder analysis showed normalization of the eGFR in 68.8–89.1% of those in CKD stages 1–3. The role of agalsidase alfa in preserving renal function is also supported by pooled data from three prospective, randomized, placebo-controlled trials and their open-label extension studies involving 108 adult male patients treated for 1–4 years [56]. The rate of eGFR loss in patients with a baseline eGFR of 30–135 ml/min per 1.73 m^2^ was less than that seen during the placebo period. Multivariate analyses revealed that the eGFR and proteinuria (<1 or ≥1 g/day) at baseline significantly predicted the rate of decline of the eGFR during treatment (Figure 4).

Renal function was evaluated in 151 men and 62 women from the Fabry Registry receiving agalsidase beta at 1 mg/kg/2 weeks for at least 2 years [51]. Multivariate logistic regression analyses were used to identify factors associated with the progression of renal disease. The risk factor most strongly associated with such progression was an averaged urinary protein:creatinine ratio ≥1 g/g and delays in treatment initiation compared with symptom onset.

In the latest long-term study on ERT effectiveness, renal function was assessed in 208 patients (mean time on ERT, 7.4 years). The mean yearly change in eGFR was -2.2 ml/min/1.73 m^2^ in men and -0.7 ml/min/1.73 m^2^ in women [62]. In another study by Tondel et al., which monitored the effects of ERT in 12 patients over 5 years using serial renal biopsies, ERT was associated with the clearance of Gb_3_ deposition from glomerular cells, especially in those receiving high doses of ERT. However, this study was carried out in a small number of patients and these results need to be confirmed in a larger study [32]. It also has been suggested that clearance of Gb_3_ from podocytes may be affected by heterogeneity between patients in the expression levels of receptors (mannose-6-phosphate receptor, sortilin and megalin) that are responsible for the uptake of alpha-galactosidase A into the lysosomes of podocytes [71].

### Early intervention with ERT may preserve renal function

Patients with impaired renal function have a less favourable outcome than those without renal impairment. A 2-year, open-label study of 26 patients with Fabry disease treated with agalsidase beta (1 mg/kg/2 weeks) reported that 9 patients experienced 12 endpoints, including 2 deaths [72]. All clinical endpoints (cerebrovascular and cardiac events, renal failure, and death) occurred in patients with impaired renal function. Despite ERT, renal function deteriorated and left ventricular posterior wall thickness (PWT) was unaltered. In contrast, patients without renal impairment at baseline had a more favourable outcome post-treatment (no clinical events, renal function remained unchanged, and PWT decreased significantly).

Similarly, in a multivariate logistic regression analysis of renal function from 151 men and 62 women from the Fabry Registry who received agalsidase beta (1 mg/kg/2 weeks) for at least 2 years, adults with Fabry disease were at risk of progressive reduction in the eGFR despite ERT if the urinary protein:creatinine ratio was ≥1 g/g [51]. Men with little urinary protein excretion and those who received ERT sooner after symptom onset had stable renal function.

The findings from these studies suggest that there is a strong case for early intervention because ERT seems less effective in more advanced disease [51,72]. Two studies evaluating the occurrence of significant negative clinical events (renal, cardiac or neural) provide further support for starting ERT early. In a prospective study of 75 patients undergoing ERT, prolonged ERT delayed the occurrence of complications [65]. The difference between treated and non-treated patients only became obvious after at least 4 years of ERT, which suggests that the risk of complications decreases with increasing treatment duration. These data also indicated that in patients affected by Fabry disease-related complications, ERT is unlikely to modify outcome. In another prospective study of 40 patients undergoing ERT and 40 matched untreated patients, no differences in the occurrence of renal, cardiac, or neural complications were observed between the cohorts. The authors suggested that a long period of ERT is required to evaluate the results but that in advanced stages of the disease ERT can improve symptoms but not slow down the progression. However, from a nephrological point of view, it is worth noting that the incidence of renal failure was lower in the ERT cohort than in the comparator cohort (10% versus 28%, respectively) [66].

The renal effects of ERT in different disease stages have also been analysed in a meta-analysis of six studies with a mean follow-up of 5 years [67]. This analysis demonstrated a smaller mean decline in renal function in males treated with ERT than in untreated males when the GFR was <60 ml/min/1.73 m^2^. However, in males with a baseline GFR of >60 ml/min/1.73 m^2^ and in females the decline in renal function was comparable between the ERT and untreated groups.

Although there is limited long-term, controlled evidence of a clinical benefit of ERT on renal function, we feel that there is adequate, less rigorous evidence to indicate that ERT is of benefit, especially when started early in the course of the disease. However, to optimize the results from ERT, it should be used, when appropriate, with other adjuvant therapies.

### Long-term safety of ERT and antibody formation

Long-term ERT can be associated, mostly in male patients, with the occurrence of antibodies against the infused enzyme [73]. While there are problems in interpreting antibody levels and comparing them between the two products due to differences in measurement methodologies and “cut off” points [73], some potentially useful data are available. IgG antibodies occur with both products, but seem to be generally higher with agalsidase beta [73]. In an open-label extension study of agalsidase beta, infusion-related reactions (rigors, temperature-change sensation, fever, nausea, headache and nasal congestion) were the most common enzyme-related adverse events [74]. With the exception of one patient, all the reactions were mild and decreased over time, similar to observations reported from the FOS registry 5-year follow up [68]. The initial increase in frequency of infusion-related reactions seems to parallel the seroconvertion rate [41]. Although high antibody titres to either product could have an inhibitory effect on agalsidase A activity *in vitro*, the relationship to clinical response is much more difficult to assess [73]. Most studies reported so far have looked at the relationship between antibody titres and surrogate markers such as Gb_3_ or lyso-Gb_3_ in urine or plasma [73,75]. Therefore, although the long-term effects of antibodies on ERT have yet to be determined, the prescribing nephrologist should be aware of the issues and potential problems.

### Dosage of ERT

The optimal dosage and regimen for ERT in Fabry disease are unclear. The licensed doses for agalsidase alfa and agalsidase beta are 0.2 mg/kg/2 weeks and 1 mg/kg/2 weeks, respectively. Clearance of Gb_3_ from podocytes has been shown to be dose-dependent, with higher doses associated with greater clearance [32]. Regarding frequency of dosing, in patients demonstrating a continuing decline in renal function, switching from bi-weekly dosing to weekly dosing of agalsidase alfa (0.2 mg/kg) has been found to improve eGFR, with the mean rate of change in eGFR slowing from -8.0 ml/min per 1.73 m^2^/year to -3.3 ml/min per 1.73 m^2^/year [69]. A recent short-term study assessing three doses of agalsidase alfa (0.2 mg every 2 weeks; 0.1 mg and 0.2 mg every week) showed a trend for the higher dose to be most effective suggesting a longer-term study was needed [76]. A study of agalsidase beta involving 21 male patients receiving the standard dose of 1 mg/kg body weight for 6 months followed by a reduced dose of 0.3 mg/kg body weight for a further 18 months showed that 100% of patients cleared Gb_3_ by 6 months while only 70% were still clear at 18 months [77].

From June 2009 to 2012, production and quality issues led to a worldwide shortage of agalsidase beta resulting in adult patients receiving reduced doses of agalsidase beta (approved dose 1 mg/kg/2 weeks, reduced dose 0.3-0.5 mg/kg/2 weeks), or switching to agalsidase alfa (administered dose 0.2 mg/kg/2 weeks) [78]. In 2010, the European Medicines Agency (EMA) focused their attention on patients treated with lower doses of agalsidase beta noting an increase in adverse events in these patients; specifically *“a pattern of adverse events resembles the natural, but accelerated, course of Fabry disease”*[78]. The effectiveness of specific ERT doses has also been evaluated in groups of patients switched from agalsidase beta to agalsidase alfa during this period. In 2011, Smid et al. reported that there were no relevant clinical differences in patients treated with variable doses of beta and/or alfa agalsidase, although both patients switching to agalsidase alfa (following a dose reduction of agalsidase beta) and those who continued on a reduced agalsidase beta dose had increased levels of lyso-Gb_3_ suggesting increased disease activity [79]. This study also showed a minimal but significant decrease in two of the quality of life subscales in the SF-36 questionnaire. End-organ damage and clinical symptoms during dose reduction of agalsidase beta or switch to agalsidase alfa has also been assessed in an observational study (N = 105) [80]. During a median follow-up of 12 months, predefined measures of organ function were stable in those continuing on agalsidase beta 1 mg/kg/2 weeks. In contrast, there was a significant decrease in renal function as assessed by eGFR in those receiving a reduced dose of agalsidase beta, as well as an increase in the number of patients experiencing pain attacks or pain crises. There was also an increase in microabuminuria and Fabry disease-related symptoms (pain aggravation and gastrointestinal symptoms) among patients switching to agalsidase alfa. Considering all data published on the clinical effects of agalsidase alfa, it is surprising how in this report the patients treated with agalsidase alfa had such a significant worsening of their symptoms in such a short time.

In other studies from Japan [81] and Italy [82] no clinical and/or laboratory differences (Gb_3_ levels) were observed after the switch from agalsidase beta to agalsidase alfa. Similar results were reported for a cohort of 40 patients in Australia although lower energy levels were observed in the male patients [83]. It is important to emphasize that all the studies on switching from agalsidase beta to agalsidase alfa were carried out in a small number of patients and for short periods of time (<2 years).

### Adjunctive therapy for proteinuria and cardiovascular disease

Reduction of proteinuria using drugs inhibiting the RAS is an essential and potent tool for improving renal outcome in patients with Fabry disease with CKD. Angiotensin-converting enzyme (ACE) inhibitors and angiotensin-II receptor antagonists (ARBs), have been demonstrated to be renoprotective in chronic nephropathy [37]. Protein levels can be reduced with ACE inhibitors/ARBs to <0.5 g/day, and are associated with significant stabilization of renal glomerular function [36,84]. Care should be taken to titrate doses to avoid hypotension [34,51,52]. The additional cardioprotective action of RAS inhibitors provides a further rationale for their use in patients with CKD [37]. These drugs will help control BP and are important for preserving renal function. Management should also include lipid-lowering and anti-platelet agents as indicated, together with appropriate prophylaxis against stroke [38]. Additionally, since cardiac arrhythmias do not respond to ERT, care should be taken to detect arrhythmias, as such events can lead to sudden cardiac death, particularly in women [9,85].

### Dialysis and transplantation

Survival of patients on dialysis with Fabry disease and ESRD is poor. Three-year survival has been shown to be 60% in Europe [86] compared with 63% in the USA [87]. Although this was better than 3-year survival in a diabetic control population (53%), it was significantly lower than in the non-diabetic control group (74%) [87]. This is almost certainly due to the added disease burden from the cardiovascular complications of Fabry disease [88]. Thus, ERT should be considered on an individual patient basis in an attempt to reduce the risk of cardiovascular events.

Renal transplantation was initially considered as a potential treatment for alleviating some of the symptoms of Fabry disease by providing a source of alpha-galactosidase A enzyme. However, this was found not to be the case, with circulating alpha-galactosidase A levels remaining low after transplantation [89]. Nevertheless, the results of renal transplantation in patients with Fabry disease are excellent: 5-year graft survival has been reported as 74%, which was superior to that of patients with ESRD due to other causes (69%), but comparable with a matched cohort [90]. Patient survival at 5 years was 81%, which was slightly lower than that of the matched cohort (90%) [90]. The results of this study were comparable with those of an earlier study, in which 5-year graft survival and 5-year patient survival were 75% and 83%, respectively [27]. In fact, the results of renal transplantation are so encouraging that it should be considered as the first-line option to correct renal dysfunction in patients with Fabry disease and ESRD [91]. However, despite the positive data supporting renal transplantation, Fabry disease has been shown to confer a higher risk of death versus diabetic control patients (odds ratio 2.15) [88].

There are few data on ERT in patients with ESRD, either on dialysis or with a functioning renal transplant. However, ERT administered to patients with ESRD on dialysis or with a history of renal transplantation has demonstrated a favourable safety profile and the same dosing regimen can be used as for patients with Fabry disease without ESRD [92]. Furthermore, there is evidence that ERT can be administered during haemodialysis, without loss of the enzyme in the dialysate [93].

### Management of Fabry disease in women

Organ involvement occurs later in life and with a lower prevalence in women than in men, but the signs and symptoms of Fabry disease should be treated in women because organ involvement is progressive and causes significant disease burden [5]. Few published studies have been dedicated specifically to women with Fabry disease [94]. However, a 4-year study of agalsidase alfa 0.2 mg/kg/2 weeks in 36 symptomatic women (mean age, 47 years) highlighted its long-term effectiveness [95].

## Areas for further research

### Chaperon molecules

An investigational therapeutic strategy, which is currently being explored, is the use of chaperon molecules to stabilize the chemical structure of the residual agalsidase alfa or to prolong the half-life of the infused enzyme [96,97]. At present, clinical studies in Fabry patients are very limited and as yet no licensed chaperone drugs are available [98].

### Biomarkers

Biomarkers have an important role in the assessment of disease activity and response to treatment in other lysosomal storage diseases. However, despite encouraging research, a biomarker has yet to be identified that is relevant to clinical outcomes in Fabry disease [67,75,99]. Nevertheless, biomarkers may play an important part as indicators of diagnosis and when to commence therapy in Fabry disease patients. They may also be useful in determining the progression of Fabry disease. Urinary protein and albumin excretion are the most important biomarkers of Fabry disease nephropathy [36,44]. Urinary Gb_3_ levels are consistently elevated in most patients with Fabry disease (although marked inter-patient variability has been observed). Urinary Gb_3_ levels decrease during ERT in men, women, and children with Fabry disease. However, despite this response, urinary Gb_3_ cannot be considered to be a suitable biomarker of clinical efficacy because baseline levels do not correlate with disease severity, and do not predict clinical efficacy [100].

Recently, plasma [101] and urinary [102] lyso-Gb_3_ have been identified as new potential biomarkers. In a study of 10 male and 8 female patients with Fabry disease, plasma lyso-Gb_3_ was increased in males, and was higher in cases of the classic form of Fabry disease than in variant Fabry hemizygotes [101]. In females, plasma lyso-Gb_3_ was moderately increased in symptomatic and asymptomatic cases, with a correlation between the increase in plasma lyso-Gb_3_ and decrease in alpha-galactosidase A activity. Lyso-Gb_3_ analogues were also isolated in the urine of 63 patients with Fabry disease [102]. Increased urinary excretion of lyso-Gb_3_ in patients with Fabry disease correlated well with several indicators of disease severity, and may be a reliable independent biomarker for the clinically important characteristics of Fabry disease. However, there was no correlation between urinary lyso-Gb_3_ and eGFR. Therefore, one cannot assume that lyso-Gb_3_ is a reliable biomarker of renal involvement [101].

In 2012, Rombach et al. confirmed elevated levels of lyso-Gb_3_ in the plasma and/or urine of patients with Fabry disease and described a reduction of plasma levels of lyso-Gb_3_ after 1 year of ERT [75]. This decline in lyso-Gb_3_ was associated with a reduction in left ventricular mass in females and a lower risk of developing white matter lesions in the nervous system for both sexes [75]. Recently, new potential biomarkers, Gb_3_ analogues, have been identified in the plasma of untreated male Fabry disease patients, but their clinical meaning still has to be validated [103].

The utility of Cystatin C as a biomarker in Fabry disease has also been evaluated [104]. In an observational, multicentre study of 89 patients (n = 42 females; n = 47 males) with Fabry disease and varying degrees of disease severity, serum Cystatin C levels were measured. The authors concluded that Cystatin C is a good and cost-effective prognostic indicator of early renal dysfunction and/or heart failure in Fabry disease [104].

Urinary uromodulin excretion has been examined in 15 male patients with Fabry disease [105]. In untreated patients, changes ranged from a normal to marked decrease or absence of urinary uromodulin frequently accompanied by aberrantly processed uromodulin. These patterns normalized in all patients on ERT and some on substrate reduction therapy. This suggests that uromodulin may also be a potential biochemical marker of renal therapeutic response to ERT.

Urine proteomic analysis based on capillary electrophoresis coupled to mass spectrometry has recently been used to identify a biomarker profile in patients with Fabry disease [106]. In 35 treatment-naïve adult female patients with Fabry disease an abnormal urine profile was identified, which was almost completely corrected following ERT [106]. A further two protein biomarkers have been identified in the urine of children with Fabry disease and type-1 diabetes using label-free quantitative proteomics. Prosaposin and ganglioside GM_2_ activator protein were significantly elevated in patients with Fabry disease and ERT was associated with a significant reduction in urinary excretion of these proteins. Therefore, these urinary biomarker models may useful as diagnostic tools for patients with Fabry disease, as well as for monitoring response to ERT [107]. However, there is still work to be done to identify a biomarker that truly reflects disease activity and progression, as well as enabling responses to ERT to be measured.

## Conclusions

Fabry disease should be considered in the differential diagnosis of proteinuria of uncertain origin to ensure an early diagnosis. Proteinuria is a risk factor for the progression of renal disease and should be managed appropriately. Early detection of renal involvement should be achieved by regular measurement of GFR and urine protein excretion in all patients, male and female, using renal biopsy where indicated. Biomarkers play an important part in the assessment of disease activity and response to treatment in other lysosomal storage diseases, and could potentially aid the diagnosis and management of Fabry renal disease.

Early intervention with ERT may help stabilize renal function or slow its decline, when used as part of a comprehensive management strategy to prevent complications of Fabry disease. Although there are currently limited long-term, controlled effectiveness data, ERT with agalsidase alfa or beta has been shown to reduce the decline in renal function in short- and long-term studies in male, female, and paediatric patients.

## Abbreviations

ACE: Angiotensin-converting enzyme; ARBs: Angiotensin-II receptor antagonists; BP: Blood pressure; CHO: Chinese hamster ovary; CKD: Chronic kidney disease; CKD-EPI: Chronic Kidney Disease Epidemiology Collaboration; ESRD: End-stage renal disease; ERT: Enzyme replacement therapy; eGFR: Estimated glomerular filtration rate; FIPI: Fabry international prognostic index; FOS: Fabry outcome survey; Gb3: Globotriaosylceramide; lyso-Gb3: Globotriaosylsphingosine; PWT: Modified diet in posterior wall thickness; MDRD: Modification of Diet in Renal Disease; RAS: Renin–angiotensin system; RRT: Renal replacement therapy.

## Competing interests

Stephen Waldek has received speaker fees, travel grants, and research support from Shire and Genzyme. Sandro Feriozzi has received speaker fees, travel grants, and research support from Shire and Genzyme.

## Authors’ contributions

SF and SW contributed to the writing of the manuscript, ensuring the inclusion of up-to-date literature. Both authors commented on all drafts and approved the final version of the manuscript.

## Authors’ information

Stephen Waldek retired in November 2011 and is working as an independent medical consultant.

## Pre-publication history

The pre-publication history for this paper can be accessed here:

http://www.biomedcentral.com/1471-2369/15/72/prepub

## References

[B1] Desnick RJ Ioannou YA Eng CM Scriver CR, Beaudet AL, Sly WS, Valle D α-Galactosidase A deficiency: Fabry disease The metabolic and molecular bases of inherited disease 2001 8 New York, USA: McGraw Hill 3733 3774

[B2] Germain DP Fabry disease Orphanet J Rare Dis 2010 5 30 10.1186/1750-1172-5-30 21092187 PMC3009617

[B3] Eng CM Fletcher J Wilcox WR Waldek S Scott CR Sillence DO Breunig F Charrow J Germain DP Nicholls K Banikazemi M Fabry disease: baseline medical characteristics of a cohort of 1765 males and females in the Fabry Registry J Inherit Metab Dis 2007 30 184 192 10.1007/s10545-007-0521-2 17347915

[B4] Hopkin RJ Bissler J Banikazemi M Clarke L Eng CM Germain DP Lemay R Tylki-Szymanska A Wilcox WR Characterization of Fabry disease in 352 pediatric patients in the Fabry Registry Pediatr Res 2008 64 550 555 10.1203/PDR.0b013e318183f132 18596579

[B5] Wilcox WR Oliveira JP Hopkin RJ Ortiz A Banikazemi M Feldt-Rasmussen U Sims K Waldek S Pastores GM Lee P Eng CM Marodi L Stanford KE Breunig F Wanner C Warnock DG Lemay RM Germain DP Females with Fabry disease frequently have major organ involvement: lessons from the Fabry Registry Mol Genet Metab 2008 93 112 128 10.1016/j.ymgme.2007.09.013 18037317

[B6] Tondel C Bostad L Hirth A Svarstad E Renal biopsy findings in children and adolescents with Fabry disease and minimal albuminuria Am J Kidney Dis 2008 51 767 776 10.1053/j.ajkd.2007.12.032 18436087

[B7] Deegan PB Baehner AF Barba Romero MA Hughes DA Kampmann C Beck M Natural history of Fabry disease in females in the Fabry outcome survey J Med Genet 2006 43 347 352 16227523 10.1136/jmg.2005.036327 PMC2563231

[B8] Ortiz A Oliveira JP Waldek S Warnock DG Cianciaruso B Wanner C Nephropathy in males and females with Fabry disease: cross-sectional description of patients before treatment with enzyme replacement therapy Nephrol Dial Transplant 2008 23 1600 1607 10.1093/ndt/gfm848 18175781

[B9] Weidemann F Niemann M Beer M Breunig F Wanner C Interdisciplinary approach towards female patients with Fabry disease Eur J Clin Invest 2012 42 455 462 10.1111/j.1365-2362.2011.02614.x 22049975

[B10] Ortiz A Cianciaruso B Cizmarik M Germain DP Mignani R Oliveira JP Villalobos J Vujkovac B Waldek S Wanner C Warnock DG End-stage renal disease in patients with Fabry disease: natural history data from the Fabry Registry Nephrol Dial Transplant 2010 25 769 775 10.1093/ndt/gfp554 19846394

[B11] Faraggiana T Churg J Grishman E Strauss L Prado A Bishop DF Schuchman E Desnick RJ Light- and electron-microscopic histochemistry of Fabry’s disease Am J Pathol 1981 103 247 262 6786101 PMC1903824

[B12] Gubler MC Lenoir G Grunfeld JP Ulmann A Droz D Habib R Early renal changes in hemizygous and heterozygous patients with Fabry’s disease Kidney Int 1978 13 223 235 10.1038/ki.1978.32 418264

[B13] Alroy J Sabnis S Kopp JB Renal pathology in Fabry disease J Am Soc Nephrol 2002 13 Suppl 2 134 138 12068025

[B14] Fischer EG Moore MJ Lager DJ Fabry disease: a morphologic study of 11 cases Mod Pathol 2006 19 1295 1301 10.1038/modpathol.3800634 16799480

[B15] Sessa A Toson A Nebuloni M Pallotti F Giordano F Battini G Maglio A Meroni M Calconi G Bertolone G Gatti P Renal ultrastructural findings in Anderson-Fabry disease J Nephrol 2002 15 109 112 12018625

[B16] Najafian B Svarstad E Bostad L Gubler MC Tondel C Whitley C Mauer M Progressive podocyte injury and globotriaosylceramide (GL-3) accumulation in young patients with Fabry disease Kidney Int 2011 79 663 670 10.1038/ki.2010.484 21160462 PMC3640823

[B17] Sessa A Meroni M Battini G Maglio A Brambilla PL Bertella M Nebuloni M Pallotti F Giordano F Bertagnolio B Tosoni A Renal pathological changes in Fabry disease J Inherit Metab Dis 2001 24 Suppl 2 66 70 11758681 10.1023/a:1012423924648

[B18] Finn L Jennette JC, Olsen JL, Schwartz MM, Silva FG Renal disease caused by familial metabolic and hematologic diseases Heptinstall’s pathology of the kidney 2007 6 Philadelphia, PA: Lippincott Williams & Wilkins 1199 1256

[B19] Sanchez-Niño MD Sanz AB Carrasco S Saleem MA Mathieson PW Valdivielso JM Ruiz-Ortega M Egido J Ortiz A Globotriaosylsphingosine actions on human glomerular podocytes: implications for Fabry nephropathy Nephrol Dial Transplant 2011 26 1797 1802 10.1093/ndt/gfq306 20504837

[B20] Valbuena C Carvalho E Bustorff M Ganhao M Relvas S Nogueira R Carneiro F Oliveira JP Kidney biopsy findings in heterozygous Fabry disease females with early nephropathy Virchows Arch 2008 453 329 338 10.1007/s00428-008-0653-2 18769939

[B21] Safyan R Whybra C Beck M Elstein D Altarescu G An association study of inflammatory cytokine gene polymorphisms in Fabry disease Eur Cytokine Netw 2006 17 271 275 17353161

[B22] Shen JS Meng XL Moore DF Quirk JM Shayman JA Schiffmann R Kaneski CR Globotriaosylceramide induces oxidative stress and up-regulates cell adhesion molecule expression in Fabry disease endothelial cells Mol Genet Metab 2008 95 163 168 10.1016/j.ymgme.2008.06.016 18707907 PMC2593623

[B23] Rombach SM Twickler TB Aerts JM Linthorst GE Wijburg FA Hollak CE Vasculopathy in patients with Fabry disease: current controversies and research directions Mol Genet Metab 2010 99 99 108 10.1016/j.ymgme.2009.10.004 19900828

[B24] Fogo AB Bostad L Svarstad E Cook WJ Moll S Barbey F Geldenhuys L West M Ferluga D Vujkovac B Howie AJ Burns A Reeve R Waldek S Noel LH Grunfeld JP Valbuena C Oliveira JP Muller J Breunig F Zhang X Warnock DG Scoring system for renal pathology in Fabry disease: report of the International Study Group of Fabry Nephropathy (ISGFN) Nephrol Dial Transplant 2010 25 2168 2177 10.1093/ndt/gfp528 19833663 PMC2902894

[B25] Liebau MC Braun F Hopker K Weitbrecht C Bartels V Muller RU Brodesser S Saleem MA Benzing T Schermer B Cybulla M Kurschat CE Dysregulated autophagy contributes to podocyte damage in Fabry’s disease PLoS One 2013 8 e63506 10.1371/journal.pone.0063506 23691056 PMC3656911

[B26] Branton MH Schiffmann R Sabnis SG Murray GJ Quirk JM Altarescu G Goldfarb L Brady RO Balow JE Austin Iii HA Kopp JB Natural history of Fabry renal disease: influence of alpha-galactosidase A activity and genetic mutations on clinical course Medicine (Baltimore) 2002 81 122 138 10.1097/00005792-200203000-00003 11889412

[B27] Ojo A Meier-Kriesche HU Friedman G Hanson J Cibrik D Leichtman A Kaplan B Excellent outcome of renal transplantation in patients with Fabry’s disease Transplantation 2000 69 2337 2339 10.1097/00007890-200006150-00020 10868636

[B28] Waldek S Patel MR Banikazemi M Lemay R Lee P Life expectancy and cause of death in males and females with Fabry disease: findings from the Fabry Registry Genet Med 2009 11 790 796 10.1097/GIM.0b013e3181bb05bb 19745746

[B29] Laney DA Bennett RL Clarke V Fox A Hopkin RJ Johnson J O’Rourke E Sims K Walter G Fabry disease practice guidelines: recommendations of the National Society of Genetic Counselors J Genet Couns 2013 22 555 564 10.1007/s10897-013-9613-3 23860966

[B30] Caudron E Dermain DP Prognon P Fabry disease: enzymatic screening using dried blood spots on filter paper Rev Med Interne 2010 31 S263 S269 21211677 10.1016/S0248-8663(10)70025-4

[B31] Selvarajah M Nicholls K Hewitson TD Becker GJ Targeted urine microscopy in Anderson-Fabry disease: a cheap, sensitive and specific diagnostic technique Nephrol Dial Transplant 2011 26 3195 3202 10.1093/ndt/gfr084 21382994

[B32] Tondel C Bostad L Larsen KK Hirth A Vikse BE Houge G Svarstad E Agalsidase benefits renal histology in young patients with Fabry disease J Am Soc Nephrol 2013 24 137 148 10.1681/ASN.2012030316 23274955 PMC3537211

[B33] Maruyama H Takata T Tsubata Y Tazawa R Goto K Tohyama J Narita I Yoshioka H Ishii S Screening of male dialysis patients for fabry disease by plasma globotriaosylsphingosine Clin J Am Soc Nephrol 2013 8 629 636 10.2215/CJN.08780812 23307880 PMC3613961

[B34] Warnock DG Daina E Remuzzi G West M Enzyme replacement therapy and Fabry nephropathy Clin J Am Soc Nephrol 2010 5 371 378 10.2215/CJN.06900909 20007680

[B35] Lukas J Giese AK Markoff A Grittner U Kolodny E Mascher H Lackner KJ Meyer W Wree P Saviouk V Rolfs A Functional characterisation of alpha-galactosidase a mutations as a basis for a new classification system in fabry disease PLoS Genet 2013 9 e1003632 10.1371/journal.pgen.1003632 23935525 PMC3731228

[B36] Schiffmann R Warnock DG Banikazemi M Bultas J Linthorst GE Packman S Sorensen SA Wilcox WR Desnick RJ Fabry disease: progression of nephropathy, and prevalence of cardiac and cerebrovascular events before enzyme replacement therapy Nephrol Dial Transplant 2009 24 2102 2111 10.1093/ndt/gfp031 19218538 PMC2698092

[B37] Schieppati A Remuzzi G Proteinuria and its consequences in renal disease Acta Paediatr Suppl 2003 92 9 13 14989459 10.1111/j.1651-2227.2003.tb00213.x

[B38] National Health Services SOP for Anderson-Fabry disease http://www.webarchive.org.uk/wayback/archive/20130325153347/http://www.specialisedservices.nhs.uk/library/23/SOP_for_Anderson_Fabry_disease.pdf

[B39] Rombach SM Baas MC ten Berge IJM Krediet RT Bemelman FJ Hollak CEM The value of estimated GFR in comparison to measured GFR for the assessment of renal function in adult patients with Fabry disease Nephrol Dial Transplant 2010 25 2549 2556 10.1093/ndt/gfq108 20215390

[B40] Schwartz GJ Munoz A Schneider MF Mak RH Kaskel F Warady BA Furth SL New equations to estimate GFR in children with CKD J Am Soc Nephrol 2009 20 629 637 10.1681/ASN.2008030287 19158356 PMC2653687

[B41] Germain DP Waldek S Banikazemi M Bushinsky DA Charrow J Desnick RJ Lee P Loew T Vedder AC Abichandani R Wilcox WR Guffon N Sustained, long-term renal stabilization after 54 months of agalsidase beta therapy in patients with Fabry disease J Am Soc Nephrol 2007 18 1547 1557 10.1681/ASN.2006080816 17409312

[B42] Schiffmann R Ries M Timmons M Flaherty JT Brady RO Long-term therapy with agalsidase alfa for Fabry disease: safety and effects on renal function in a home infusion setting Nephrol Dial Transplant 2006 21 345 354 10.1093/ndt/gfi152 16204287

[B43] Wanner C Oliveira JP Ortiz A Mauer M Germain DP Linthorst GE Serra AL Marodi L Mignani R Cianciaruso B Vujkovac B Lemay R Beitner-Johnson D Waldek S Warnock DG Prognostic indicators of renal disease progression in adults with Fabry disease: natural history data from the Fabry Registry Clin J Am Soc Nephrol 2010 5 2220 2228 10.2215/CJN.04340510 20813854 PMC2994083

[B44] Banikazemi M Bultas J Waldek S Wilcox WR Whitley CB McDonald M Finkel R Packman S Bichet DG Warnock DG Desnick RJ Agalsidase-beta therapy for advanced Fabry disease: a randomized trial Ann Intern Med 2007 146 77 86 10.7326/0003-4819-146-2-200701160-00148 17179052

[B45] Mehta A Ricci R Widmer U Dehout F Garcia de Lorenzo A Kampmann C Linhart A Sunder-Plassmann G Ries M Beck M Fabry disease defined: baseline clinical manifestations of 366 patients in the Fabry Outcome Survey Eur J Clin Invest 2004 34 236 242 10.1111/j.1365-2362.2004.01309.x 15025684

[B46] Chen HC Tsai JH Lai YH Guh JY Renal changes in heterozygous Fabry’s disease – a family study Am J Kidney Dis 1990 15 180 183 2105640 10.1016/s0272-6386(12)80518-x

[B47] Sheu SS Chan LP Liao SC Hsiao KJ Shu KH Lu YS Cheng CH Lian JD Fabry’s disease: clinical, pathologic and biochemical manifestations in two Chinese males Zhonghua Yi Xue Za Zhi (Taipei) 1994 54 368 372 7834562

[B48] Ries M Bettis KEB Choyke P Kopp JB Austin HA Brady RO Schiffmann R Parapelvic kidney cysts: a distinguishing feature with high prevalence in Fabry disease Kidney Int 2004 66 978 982 10.1111/j.1523-1755.2004.00846.x 15327390

[B49] Kleinert J Dehout F Schwarting A de Lorenzo AG Ricci R Kampmann C Beck M Ramaswami U Linhart A Gal A Houge G Widmer U Mehta A Sunder-Plassmann G Prevalence of uncontrolled hypertension in patients with Fabry disease Am J Hypertens 2006 19 782 787 10.1016/j.amjhyper.2006.01.011 16876675

[B50] Jain G Warnock DG Blood pressure, proteinuria and nephropathy in Fabry disease Nephron Clin Pract 2011 118 43 48 10.1159/000320903 21071972

[B51] Warnock DG Ortiz A Mauer M Linthorst GE Oliveira JP Serra AL Marodi L Mignani R Vujkovac B Beitner-Johnson D Lemay R Cole JA Svarstad E Waldek S Germain DP Wanner C Renal outcomes of agalsidase beta treatment for Fabry disease: role of proteinuria and timing of treatment initiation Nephrol Dial Transplant 2012 27 1042 1049 10.1093/ndt/gfr420 21804088 PMC3289896

[B52] Fervenza FC Torra R Lager DJ Fabry disease: an underrecognized cause of proteinuria Kidney Int 2008 73 1193 1199 10.1038/sj.ki.5002677 18033242

[B53] Rozenfeld P Neumann PM Treatment of Fabry disease: current and emerging strategies Curr Pharm Biotechnol 2011 12 916 922 10.2174/138920111795542705 21235448

[B54] Lidove O Joly D Barbey F Bekri S Alexandra JF Peigne V Jaussaud R Papo T Clinical results of enzyme replacement therapy in Fabry disease: a comprehensive review of literature Int J Clin Pract 2007 61 293 302 10.1111/j.1742-1241.2006.01237.x 17263716

[B55] Schiffmann R Kopp JB Austin HA Sabnis S Moore DF Weibel T Balow JE Brady RO Enzyme replacement therapy in Fabry disease: a randomized controlled trial JAMA 2001 285 2743 2749 10.1001/jama.285.21.2743 11386930

[B56] West M Nicholls K Mehta A Clarke JTR Steiner R Beck M Barshop BA Rhead W Mensah R Ries M Schiffmann R Agalsidase alfa and kidney dysfunction in Fabry disease J Am Soc Nephrol 2009 20 1132 1139 10.1681/ASN.2008080870 19357250 PMC2678048

[B57] Mehta A Clarke JT Giugliani R Elliott P Linhart A Beck M Sunder-Plassmann G Natural course of Fabry disease: changing pattern of causes of death in FOS – Fabry Outcome Survey J Med Genet 2009 46 548 552 10.1136/jmg.2008.065904 19473999

[B58] Hughes DA Malmenas M Deegan PB Elliott PM Ginsberg L Hajioff D Ioannidis AS Orteu CH Ramaswami U West M Pastores GM Jenkinson C Fabry International Prognostic Index: a predictive severity score for Anderson-Fabry disease J Med Genet 2012 49 212 220 10.1136/jmedgenet-2011-100407 22315436

[B59] Hughes DA Ramaswami U Barba Romero MÃ Deegan P Age adjusting severity scores for Anderson-Fabry disease Mol Genet Metab 2010 101 219 227 10.1016/j.ymgme.2010.06.002 20691627

[B60] Giannini EH Mehta AB Hilz MJ Beck M Bichet DG Brady RO West M Germain DP Wanner C Waldek S Clarke JT Mengel E Strotmann JM Warnock DG Linhart A A validated disease severity scoring system for Fabry disease Mol Genet Metab 2010 99 283 290 10.1016/j.ymgme.2009.10.178 19951842

[B61] Eng CM Guffon N Wilcox WR Germain DP Lee P Waldek S Caplan L Linthorst GE Desnick RJ Safety and efficacy of recombinant human alpha-galactosidase A – replacement therapy in Fabry’s disease N Engl J Med 2001 345 9 16 10.1056/NEJM200107053450102 11439963

[B62] Feriozzi S Torras J Cybulla M Nicholls K Sunder-Plassmann G West M The effectiveness of long-term agalsidase alfa therapy in the treatment of Fabry nephropathy Clin J Am Soc Nephrol 2012 7 60 69 10.2215/CJN.03130411 22246281 PMC3265340

[B63] Schwarting A Dehout F Feriozzi S Beck M Mehta A Sunder-Plassmann G Enzyme replacement therapy and renal function in 201 patients with Fabry disease Clin Nephrol 2006 66 77 84 16939062

[B64] Pisani A Visciano B Roux GD Sabbatini M Porto C Parenti G Imbriaco M Enzyme replacement therapy in patients with Fabry disease: state of the art and review of the literature Mol Genet Metab 2012 107 267 275 10.1016/j.ymgme.2012.08.003 22963910

[B65] Rombach SM Smid BE Bouwman MG Linthorst GE Dijkgraaf MG Hollak CE Long term enzyme replacement therapy for Fabry disease: effectiveness on kidney, heart and brain Orphanet J Rare Dis 2013 8 47 10.1186/1750-1172-8-47 23531228 PMC3626869

[B66] Weidemann F Niemann M Störk S Breunig F Beer M Sommer C Herrmann S Ertl G Wanner C Long-term outcome of enzyme-replacement therapy in advanced Fabry disease: evidence for disease progression towards serious complications J Intern Med 2013 274 331 341 10.1111/joim.12077 23586858 PMC4282332

[B67] Rombach SM Smid BE Linthorst GE Dijkgraaf MG Hollak CE Natural course of Fabry disease and the effectiveness of enzyme replacement therapy: a systematic review and meta-analysis : effectiveness of ERT in different disease stages J Inherit Metab Dis 2014 [Epub ahead of print] 10.1007/s10545-014-9677-8 24492980

[B68] Mehta A Beck M Elliott P Giugliani R Linhart A Sunder-Plassmann G Schiffmann R Barbey F Ries M Clarke JTR Enzyme replacement therapy with agalsidase alfa in patients with Fabry’s disease: an analysis of registry data Lancet 2009 374 1986 1996 10.1016/S0140-6736(09)61493-8 19959221

[B69] Schiffmann R Askari H Timmons M Robinson C Benko W Brady RO Ries M Weekly enzyme replacement therapy may slow decline of renal function in patients with Fabry disease who are on long-term biweekly dosing J Am Soc Nephrol 2007 18 1576 1583 10.1681/ASN.2006111263 17409308 PMC1978101

[B70] Lindeman RD Tobin J Shock NW Longitudinal studies on the rate of decline in renal function with age J Am Geriatr Soc 1985 33 278 285 3989190 10.1111/j.1532-5415.1985.tb07117.x

[B71] Prabakaran T Nielsen R Larsen JV Sorensen SS Feldt-Rasmussen U Saleem MA Petersen CM Verroust PJ Christensen EI Receptor-mediated endocytosis of alpha-galactosidase A in human podocytes in Fabry disease PLoS One 2011 6 e25065 10.1371/journal.pone.0025065 21949853 PMC3176300

[B72] Breunig F Weidemann F Strotmann J Knoll A Wanner C Clinical benefit of enzyme replacement therapy in Fabry disease Kidney Int 2006 69 1216 1221 10.1038/sj.ki.5000208 16609685

[B73] Deegan PB Fabry disease, enzyme replacement therapy and the significance of antibody responses J Inherit Metab Dis 2012 35 227 243 10.1007/s10545-011-9400-y 22037707

[B74] Wilcox WR Banikazemi M Guffon N Waldek S Lee P Linthorst GE Desnick RJ Germain DP Long-term safety and efficacy of enzyme replacement therapy for Fabry disease Am J Hum Genet 2004 75 65 74 10.1086/422366 15154115 PMC1182009

[B75] Rombach SM Aerts JM Poorthuis BJ Groener JE Donker-Koopman W Hendriks E Mirzaian M Kuiper S Wijburg FA Hollak CE Linthorst GE Long-term effect of antibodies against infused alpha-galactosidase A in Fabry disease on plasma and urinary (lyso)Gb3 reduction and treatment outcome PLoS One 2012 7 e47805 10.1371/journal.pone.0047805 23094092 PMC3477102

[B76] Hughes DA Deegan PB Milligan A Wright N Butler LH Jacobs A Mehta AB A randomised, double-blind, placebo-controlled, crossover study to assess the efficacy and safety of three dosing schedules of agalsidase alfa enzyme replacement therapy for Fabry disease Mol Genet Metab 2013 109 269 275 10.1016/j.ymgme.2013.04.015 23702393

[B77] Lubanda JC Anijalg E Bzduch V Thurberg BL Benichou B Tylki-Szymanska A Evaluation of a low dose, after a standard therapeutic dose, of agalsidase beta during enzyme replacement therapy in patients with Fabry disease Genet Med 2009 11 256 264 10.1097/GIM.0b013e3181981d82 19265719

[B78] European Medicines Agency Assessment report on the shortage of Fabrazyme. Overview of shortage period: spontaneous reports from June 2009 through 15 September 2010 and registry data from June 2009 through 05 August 2010 http://www.ema.europa.eu/docs/en_GB/document_library/Other/2010/11/WC500099241.pdf Accessed 16-9-2013

[B79] Smid BE Rombach SM Aerts JM Kuiper S Mirzaian M Overkleeft HS Poorthuis BJ Hollak CE Groener JE Linthorst GE Consequences of a global enzyme shortage of agalsidase beta in adult Dutch Fabry patients Orphanet J Rare Dis 2011 6 69 10.1186/1750-1172-6-69 22041095 PMC3219561

[B80] Weidemann F Kramer J Duning T Lenders M Canaan-Kuhl S Krebs A Gonzalez HG Sommer C Uceyler N Niemann M Stork S Schelleckes M Reiermann S Stypmann J Brand SM Wanner C Brand E Patients with Fabry disease after enzyme replacement therapy dose reduction versus treatment switch J Am Soc Nephrol 2014 25 837 849 10.1681/ASN.2013060585 24556354 PMC3968503

[B81] Tsuboi K Yamamoto H Clinical observation of patients with Fabry disease after switching from agalsidase beta (Fabrazyme) to agalsidase alfa (Replagal) Genet Med 2012 14 779 786 10.1038/gim.2012.39 PMC3908501 22498845

[B82] Pisani A Spinelli L Visciano B Capuano I Sabbatini M Riccio E Messalli G Imbriaco M Effects of switching from agalsidase beta to agalsidase alfa in 10 patients with anderson-fabry disease JIMD Rep 2013 9 41 48 23430546 10.1007/8904_2012_177 PMC3565648

[B83] Ghali J Nicholls K Denaro C Sillence D Chapman I Goldblatt J Thomas M Fletcher J Effect of reduced agalsidase beta dosage in fabry patients: the Australian experience JIMD Rep 2012 3 33 43 23430871 10.1007/8904_2011_44 PMC3509855

[B84] Tahir H Jackson LL Warnock DG Antiproteinuric therapy and fabry nephropathy: sustained reduction of proteinuria in patients receiving enzyme replacement therapy with agalsidase-beta J Am Soc Nephrol 2007 18 2609 2617 10.1681/ASN.2006121400 17656478

[B85] Niemann M Herrmann S Hu K Breunig F Strotmann J Beer M Machann W Voelker W Ertl G Wanner C Weidemann F Differences in Fabry cardiomyopathy between female and male patients: consequences for diagnostic assessment JACC Cardiovasc Imaging 2011 4 592 601 10.1016/j.jcmg.2011.01.020 21679893

[B86] Tsakiris D Simpson HK Jones EH Briggs JD Elinder CG Mendel S Piccoli G dos Santos JP Tognoni G Vanrenterghem Y Valderrabano F Report on management of renale failure in Europe, XXVI, 1995. Rare diseases in renal replacement therapy in the ERA-EDTA Registry Nephrol Dial Transplant 1996 11 Suppl 7 4 20 9067983 10.1093/ndt/11.supp7.4

[B87] Thadhani R Wolf M West ML Tonelli M Ruthazer R Pastores GM Obrador GT Patients with Fabry disease on dialysis in the United States Kidney Int 2002 61 249 255 10.1046/j.1523-1755.2002.00097.x 11786107

[B88] Mignani R Feriozzi S Schaefer RM Breunig F Oliveira JP Ruggenenti P Sunder-Plassmann G Dialysis and transplantation in Fabry disease: indications for enzyme replacement therapy Clin J Am Soc Nephrol 2010 5 379 385 10.2215/CJN.05570809 20056752

[B89] Van den Bergh FA Rietra PJ Kolk-Vegter AJ Bosch E Tager JM Therapeutic implications of renal transplantation in a patient with Fabry’s disease Acta Med Scand 1976 200 249 256 824932

[B90] Shah T Gill J Malhotra N Takemoto SK Bunnapradist S Kidney transplant outcomes in patients with Fabry disease Transplantation 2009 87 280 285 10.1097/TP.0b013e318191a842 19155985

[B91] Cybulla M Kurschat C West M Nicholls K Torras J Sunder-Plassmann G Feriozzi S Kidney transplantation and enzyme replacement therapy in patients with Fabry disease J Nephrol 2013 26 645 651 10.5301/jn.5000214 23023720

[B92] Pastores GM Boyd E Crandall K Whelan A Piersall L Barnett N Safety and pharmacokinetics of agalsidase alfa in patients with Fabry disease and end-stage renal disease Nephrol Dial Transplant 2007 22 1920 1925 10.1093/ndt/gfm096 17395657

[B93] Kosch M Koch HG Oliveira JP Soares C Bianco F Breuning F Rasmussen AK Schaefer RM Enzyme replacement therapy administered during hemodialysis in patients with Fabry disease Kidney Int 2004 66 1279 1282 10.1111/j.1523-1755.2004.00883.x 15327428

[B94] Parini R Feriozzi S Females and children with Anderson–Fabry disease: diagnosis, monitoring, benefits of enzyme replacement therapy (ERT) and considerations on timing of starting ERT Expert Opin Orphan Drugs 2013 1 315 330 10.1517/21678707.2013.776957

[B95] Whybra C Miebach E Mengel E Gal A Baron K Beck M Kampmann C A 4-year study of the efficacy and tolerability of enzyme replacement therapy with agalsidase alfa in 36 women with Fabry disease Genet Med 2009 11 441 449 10.1097/GIM.0b013e3181a23bec 19346951

[B96] Ishii S Chang HH Yoshioka H Shimada T Mannen K Higuchi Y Taguchi A Fan JQ Preclinical efficacy and safety of 1-deoxygalactonojirimycin in mice for Fabry disease J Pharmacol Exp Ther 2009 328 723 731 10.1124/jpet.108.149054 19106170

[B97] Porto C Pisani A Rosa M Acampora E Avolio V Tuzzi MR Visciano B Gagliardo C Materazzi S la Marca G Andria G Parenti G Synergy between the pharmacological chaperone 1-deoxygalactonojirimycin and the human recombinant alpha-galactosidase A in cultured fibroblasts from patients with Fabry disease J Inherit Metab Dis 2012 35 513 520 10.1007/s10545-011-9424-3 22187137

[B98] Giugliani R Waldek S Germain DP Nicholls K Bichet DG Simosky JK Bragat AC Castelli JP Benjamin ER Boudes PF A Phase 2 study of migalastat hydrochloride in females with Fabry disease: selection of population, safety and pharmacodynamic effects Mol Genet Metab 2013 109 86 92 10.1016/j.ymgme.2013.01.009 23474038

[B99] Mehta AB Anderson-Fabry disease: developments in diagnosis and treatment Int J Clin Pharmacol Ther 2009 47 Suppl 1 66 74 10.5414/cpp47066 20040315

[B100] Mehta A West ML Pintos-Morell G Reisin R Nicholls K Figuera LE Parini R Carvalho LR Kampmann C Pastores GM Lidove O Therapeutic goals in the treatment of Fabry disease Genet Med 2010 12 713 720 10.1097/GIM.0b013e3181f6e676 20975569

[B101] Togawa T Kodama T Suzuki T Sugawara K Tsukimura T Ohashi T Ishige N Suzuki K Kitagawa T Sakuraba H Plasma globotriaosylsphingosine as a biomarker of Fabry disease Mol Genet Metab 2010 100 257 261 10.1016/j.ymgme.2010.03.020 20409739

[B102] Auray-Blais C Boutin M Gagnon R Dupont FO Lavoie P Clarke JTR Urinary globotriaosylsphingosine-related biomarkers for Fabry disease targeted by metabolomics Anal Chem 2012 84 2745 2753 10.1021/ac203433e 22309310

[B103] Manwaring V Boutin M Auray-Blais C A metabolomic study to identify new globotriaosylceramide-related biomarkers in the plasma of Fabry disease patients Anal Chem 2013 85 9039 9048 10.1021/ac401542k 23968398

[B104] Torralba-Cabeza MÃ Olivera S Hughes DA Pastores GM Mateo RN Perez-Calvo JI Cystatin C and NT-proBNP as prognostic biomarkers in Fabry disease Mol Genet Metab 2011 104 301 307 10.1016/j.ymgme.2011.06.021 21795086

[B105] Vylet’al P Hulkova H Zivna M Berna L Novak P Elleder M Kmoch S Abnormal expression and processing of uromodulin in Fabry disease reflects tubular cell storage alteration and is reversible by enzyme replacement therapy J Inherit Metab Dis 2008 31 508 517 10.1007/s10545-008-0900-3 18651238

[B106] Kistler AD Siwy J Breunig F Jeevaratnam P Scherl A Mullen W Warnock DG Wanner C Hughes DA Mischak H Wuthrich RP Serra AL A distinct urinary biomarker pattern characteristic of female Fabry patients that mirrors response to enzyme replacement therapy PLoS One 2011 6 e20534 10.1371/journal.pone.0020534 21698285 PMC3115947

[B107] Manwaring V Heywood WE Clayton R Lachmann RH Keutzer J Hindmarsh P Winchester B Heales S Mills K The identification of new biomarkers for identifying and monitoring kidney disease and their translation into a rapid mass spectrometry-based test: evidence of presymptomatic kidney disease in pediatric Fabry and type-I diabetic patients J Proteome Res 2013 12 2013 2021 10.1021/pr301200e 23464927

